# Brilacidin’s Antifungal Mechanism: Insights from Lipid Membrane Models

**DOI:** 10.3390/antibiotics15060548

**Published:** 2026-05-29

**Authors:** María Victoria López Nota Francisco, Milagro Mottola, Jessica Aye Valdivia Pérez, Julieta Tallone, Thaila Fernanda dos Reis, Gustavo H. Goldman, Candelaria Inés Cámara, Maria Laura Fanani

**Affiliations:** 1 Departamento de Química Biológica Ranwel Caputto, Facultad de Ciencias Químicas, Universidad Nacional de Córdoba, Córdoba X5000HUA, Argentina; maria.victoria.lopez@unc.edu.ar (M.V.L.N.F.); mmottola@unc.edu.ar (M.M.); jessica.perez@unc.edu.ar (J.A.V.P.); julieta.tallone@mi.unc.edu.ar (J.T.); 2 Centro de Investigaciones en Química Biológica de Córdoba (CIQUIBIC), Consejo Nacional de Investigaciones Científicas y Técnicas (CONICET), Córdoba X5000HUA, Argentina; 3 Instituto de Química, Universidade Estadual de São Paulo, Araraquara 14800-901, SP, Brazil; thaila.reis@unesp.br; 4 National Institute of Science and Technology in Human Pathogenic Fungi, Ribeirão Preto 14040-900, SP, Brazil; ggoldman@usp.br; 5 Faculdade de Ciências Farmacêuticas de Ribeirão Preto, Universidade de São Paulo, Ribeirão Preto 14040-903, SP, Brazil; 6 Departamento de Fisicoquímica, Facultad de Ciencias Químicas, Universidad Nacional de Córdoba, Córdoba X5000HUA, Argentina; candelaria_camara@unc.edu.ar; 7 Consejo Nacional de Investigaciones Científicas y Técnicas (CONICET), Instituto de Investigaciones en Fisicoquímica de Córdoba (INFIQC), Córdoba X5000HUA, Argentina

**Keywords:** aryl amide polymers, polymer dipole moment, lipid membrane insertion, black lipid membranes, polymer intrinsic fluorescence, ergosterol-dependent activity

## Abstract

**Background/Objectives:** BRI is a synthetic arylamide polymer designed to mimic the electrostatic and amphiphilic features of defensin-type antimicrobial peptides (AMPs), although its molecular organization and activity have not been experimentally validated. This study presents the first integrated computational and experimental characterization of BRI to define the physicochemical basis of its AMP-like behavior and membrane interactions. **Methods:** Molecular modelling was used to evaluate the structural and electrostatic properties of BRI. Model lipid membranes were used to study membrane interactions. Fluorescence spectroscopy, electrochemical measurements, and ζ-potential analyses were performed to characterize membrane insertion, aggregation, ionic conductance, and membrane resistance. Microbiology assays evaluating synergy with azole were also assessed. **Results:** Molecular modelling showed that BRI is a flexible molecule with cationic and hydrophobic surfaces, a strong amphiphilic dipole, and a dominant +4 charge state, explaining its sensitivity to ionic strength and membrane interactions. BRI displayed two membrane-dependent mechanisms of action. In zwitterionic phospholipid membranes, BRI resembled canonical AMPs, showing membrane insertion, pore formation, and increased ionic conductance. In anionic ergosterol-containing membranes mimicking fungal cells, BRI exhibited sterol-dependent insertion, in-plane aggregation, and modulation of membrane resistance without pore formation. Fluorescence, electrochemical, and ζ-potential measurements supported BRI–BRI interactions at the membrane interface and sensitivity to lipid domain organization. BRI also synergized with azole antifungal drugs, suggesting a mechanistic role for ergosterol in its antifungal activity. **Conclusions:** These findings reveal a sterol- and domain-mediated mechanism for arylamide polymers and identify lipid organization as a key determinant of antifungal activity. The dependence of BRI activity on ergosterol content provides a mechanistic explanation for its synergy with azole antifungals and supports further investigation of BRI as a membrane-active antifungal agent.

## 1. Introduction

Antimicrobial peptides (AMPs) are fundamental components of innate immunity in animals and plants. These small (~2 kDa), cationic, amphipathic molecules—including defensins—are characterized by a net positive charge (+1 or higher), hydrophobicity, α-helical secondary structure, and self-association capacity [1]. Because they target microbial membranes, AMPs have gained attention as potential alternatives to conventional antibiotics. Drugs targeting the bacterial membrane generally exhibit lower resistance potential compared to those acting on intracellular targets, as significant alterations to membrane composition or structure impose substantial energetic costs and compromise cell viability; however, resistance can still emerge through mechanisms such as lipid modifications, efflux pumps, and protease degradation [2].

Despite significant progress over the past three decades, the clinical application of AMPs remains limited by challenges, including host toxicity and poor stability [3]. To overcome these limitations, synthetic antimicrobial peptide mimetics (SAMPs) have been developed to reproduce the amphiphilic organization and cationic charge of natural AMPs while incorporating unnatural backbones that improve proteolytic stability and reduce production costs [1,4]. These foldable polymeric molecules—also known as foldamers—represent a promising strategy against multidrug-resistant pathogens.

Brilacidin (BRI, PMX-30063) is a synthetic arylamide foldamer designed to mimic the membrane-disruptive behavior of defensin-type AMPs [5,6]. Currently undergoing FDA fast-tracked clinical trials for oral mycosis and inflammatory bowel disease, it is also being evaluated for acute bacterial skin infections (NCT02324335; NCT01211470; NCT02052388) [7,8]. BRI exhibits broad-spectrum antimicrobial activity against Gram-positive and Gram-negative bacteria, as well as antiviral activity, including SARS-CoV-2 [9]. In vitro studies show bactericidal effects at low micromolar concentrations (0.5–1 µM) with minimal risk of resistance development. Additionally, BRI exhibits antifungal properties, particularly against *Cryptococcus neoformans*, the pathogen responsible for pulmonary cryptococcosis in humans [4,7,10].

New strategies are urgently needed to combat life-threatening fungal infections, as resistance to existing antifungal drugs continues to emerge. BRI has been identified as a potent antifungal agent against *Cryptococcus neoformans*, the causative agent of cryptococcal meningitis, and shows synergistic effects with caspofungin (CAS) against both CAS-sensitive and CAS-resistant isolates of *Aspergillus fumigatus*, *Candida albicans*, and *C. auris* [10,11]. Furthermore, BRI increases azole efficacy against *A. fumigatus* by disrupting the cell wall integrity pathway and altering membrane potential [10]. Whether BRI acts exclusively at the fungal cell surface or is also transported intracellularly remains unclear, suggesting multiple mechanisms of action. Nevertheless, the synergy between BRI and azoles, which deplete ergosterol (ERG) in the fungal plasma membrane, emphasize the importance of its interaction with membranes, suggesting that membrane composition strongly influences BRI activity.

Studies using *Saccharomyces cerevisiae* as a model system suggest that BRI affects membrane organization, the cell wall integrity pathway, and calcium metabolism [11]. In *C. neoformans*, mutants deficient in ERG and glycosphingolipid synthesis exhibit lower minimal inhibitory concentration (MIC) values for BRI, while supplementation with free ERG reverses this effect [11], highlighting the role of lipid composition in BRI’s mechanism of action. Moreover, BRI increases the permeability of *C. neoformans* membranes to propidium iodide (PI), providing direct evidence of membrane permeabilization in BRI-treated cells. Furthermore, BRI induces membrane depolarization in both fungi and bacteria [6,10].

This work aims to investigate the interaction of BRI with lipid membranes to gain deeper insight into its antifungal mechanism of action and its sensitivity to membrane composition. To this end, we used model lipid membrane systems with well-defined compositions [12] that reproduce key features of fungal plasma membranes while avoiding the experimental complexity associated with the active metabolism of living cells. We examined whether BRI reproduces AMP-like behavior [1] by evaluating its ability to associate with membranes, alter their electrostatic properties, and increase ion permeability. We also evaluated how BRI responds to lipid composition changes that mimic the membrane alterations associated with its synergistic effects with azoles in fungal cells.

## 2. Materials and Methods

### 2.1. Materials

**Figure 1:**
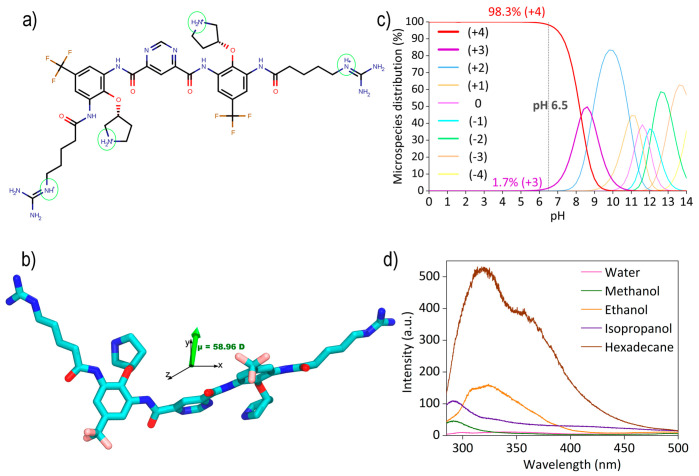
Chemical structure and physicochemical properties of Brilacidin, [4-*N*,6-*N*-bis [3-[5-(diaminomethylideneamino) pentanoylamino]-2-[(3*R*)-pyrrolidin-3-yl]oxy-5-(trifluoromethyl)phenyl] pyrimidine-4,6-dicarboxamide] (BRI). (**a**) Two-dimensional and (**b**) three-dimensional structures of BRI. Geometry optimization and electronic properties (electrostatic charges and molecular dipole moment) were calculated at the DFT level using the B3LYP/6-31+G* method with implicit solvent (PCM, water). (**c**) BRI speciation diagram as a function of pH, highlighting the predominance of the +4 charged species at pH 6.5. (**d**) Intrinsic fluorescence spectra of BRI (40 μM BRI) in solvents of different polarity.

Purified lipids 1,2-Dioleoyl-*sn*-glycero-3-phosphoethanolamine (DOPE), 1,2-Dioleoyl-*sn*-glycero-3-phosphocholine (DOPC), 1,2-dimyristoyl-*sn*-glycero-3-phosphate (DMPA), and ergosterol (ERG) were obtained from Merck KGaA (Darmstadt, Germany). Brilacidin (BRI, formerly PMX-30063, MW 936.9 Da) [4-*N*,6-*N*-bis [3-[5-(diaminomethylideneamino)pentanoylamino]-2-[(3R)-pyrrolidin-3-yl]oxy-5-(trifluoromethyl)phenyl] pyrimidine-4,6-dicarboxamide] was synthesized and donated by Dr. William F. deGrado (University of California, USA). All other reagents were of analytical grade (99% pure) and used without further purification. 6-dodecanoyl-2-dimethyl-aminonaphthalene (LAURDAN) was obtained from Invitrogen (Eugene, OR, USA). Water was purified using a Milli-Q system (Millipore, Billerica, MA, USA) to a resistivity of ~18.5 MΩ. The structure of BRI is shown in Figure 1a, and the structures of the lipids used are shown in Figure 3a.

### 2.2. BRI Parametrization and Electronic Properties

The molecular structure of BRI was obtained from the PubChem database (CID 25023695) [13]. The initial three-dimensional structure was constructed and visualized using MarvinSketch (ChemAxon, version 22.2.1) [14]. Acid–base speciation as a function of pH was evaluated using MarvinSketch tools, allowing identification of the dominant protonation states across the physiological pH range. Based on this analysis, the predominant molecular species at pH 6.5, carrying a net charge of +4, was selected for further calculations.

Quantum chemical calculations were performed to optimize the geometry and determine the electronic properties of BRI. Geometry optimization was carried out at the density functional theory (DFT) level using the B3LYP functional and the 6-31+G* basis set, as implemented in Gaussian 03 and Gaussian 09 [15]. Solvent effects were included using the polarizable continuum model (PCM) with water as the implicit solvent. From the optimized structure, electrostatic potential (ESP) calculations were performed on a Merz–Kollman grid, and atomic partial charges were derived using the restrained electrostatic potential (RESP) fitting procedure [16].

The resulting optimized geometry was used to generate a three-dimensional molecular representation of BRI and to compute key electronic descriptors, with particular emphasis on the molecular dipole moment. These calculations were performed to characterize the charge distribution and polarity of BRI at pH 6.5, which are relevant for its interaction with lipid membranes, following computational strategies previously applied to bioactive molecules interacting with model membranes [17]. Although force-field–compatible parameters were generated using AmberTools (AmberTools22, https://ambermd.org/AmberTools.php, accessed on 11 May 2026) within the General AMBER Force Field (GAFF) framework [18] and following established parametrization protocols [19,20], the resulting parametrized structure was not used for subsequent molecular dynamics simulations. Instead, the parametrization workflow was used solely to obtain a physically consistent molecular structure, charge distribution, and dipole moment under defined protonation conditions.

### 2.3. Microbiology Assay

The wild-type strain of *A. fumigatus* (reference strain A1160) was cultured in minimal medium (MM; 1% [*w*/*v*] glucose, 50 mL of 20× salt solution, 2% [*w*/*v*] trace elements, pH 6.5). For solid medium preparation, MM was supplemented with 2% agar. BRI was tested at concentrations of 20 or 40 µM, either alone or in combination with caspofungin (CAS; 0.5 µg/mL). The minimal inhibitory concentration (MIC) of BRI for this strain is >80 µM. Wells containing medium alone, CAS (0.5 µg/mL), or DMSO served as controls. Cultures were incubated at 37 °C for 120 h, after which radial growth was measured. All experiments were performed in triplicate. Statistical analysis was performed using a one-way ANOVA with Dunnett’s multiple comparisons test.

### 2.4. The Model Lipid Membrane

A lipid mixture was formulated to mimic the plasma membrane of fungi, based on the model membrane described in Ref. [21]. The synthetic membrane consisted of phosphatidylcholine (PC), phosphatidylethanolamine (PE), phosphatidic acid (PA), and ergosterol (ERG) in a 5:4:1:2 molar ratio. This composition was used for both vesicle and monolayer experiments. To construct these membranes, the unsaturated lipids DOPC and DOPE and the saturated lipid DMPA were selected, resulting in a membrane containing 16.7 mol% ERG (M2). In addition, a lipid membrane containing 8.3 mol% ERG (M1) was prepared for comparative experiments. The final compositions were as follows:

M1: DOPC/DOPE/DMPA/ERG (46.3:37:8.3:8.3)

M2: DOPC/DOPE/DMPA/ERG (41.7:33.4:8.3:16.7)

For most experiments, pure DOPC membranes were used as a control, as DOPC is a common zwitterionic phospholipid found in many cell membranes.

### 2.5. Liposome Preparation and Fluorescence Measurements

Large unilamellar vesicles (LUVs) composed of the chosen lipid mixtures were used as models of fungal plasma membranes. LUVs were formed following a previously reported method [22]. To prepare multilamellar vesicles, a uniform lipid film was first formed on the inner surface of a glass test tube by evaporating a chloroform/methanol (2:1) lipid solution (1 mM) under a stream of nitrogen. Any remaining solvent traces were removed using a high-vacuum chamber for 1 h. The dried lipid film was then hydrated with 5 mM HEPES buffer (pH 6.5) and vigorously mixed. The resulting suspension underwent nine freeze–thaw cycles, alternating between 0 °C and 40 °C. LUVs with an average diameter of 100 nm were obtained by extruding multilamellar vesicles 21 times through polycarbonate filters with 100 nm pores at room temperature.

The intrinsic fluorescence of BRI was measured by recording fluorescence spectra of BRI dissolved in different solvents or in 5 mM HEPES buffer at pH 6.5 (40 µM) in the presence of LUVs (lipid concentration 200 µM). Samples were excited at 266 nm, and emission spectra were collected from 280 to 500 nm. All fluorescence measurements were performed under steady-state conditions using a Cary Eclipse Spectrofluorometer (Agilent Technologies, Melbourne, Australia) equipped with a temperature-regulated multicell holder at 25 °C and a 3 mm optical path-length cell. All experiments were performed in triplicate.

### 2.6. Particle Analysis

Particle-size distributions were assessed by dynamic light scattering (DLS) at varying peptide concentrations using a Nicomp™ 380 Submicron Particle Sizer (Entegris, Santa Barbara, CA, USA), equipped with a 530 nm laser. Measurements were performed at a scattering angle of 90°. The hydrodynamic diameter of the particles was calculated using the Stokes–Einstein equation. ζ-potential measurements were carried out using a Zetasizer SZ-100-Z (Horiba, Ltd., Kyoto, Japan), equipped with a solid-state semiconductor laser (532 nm, 10 mW), employing the laser-Doppler velocimetry technique. BRI adsorption to lipid vesicles was analyzed by fitting ζ-potential vs. BRI concentration plots using the following sigmoidal (Hill-type) equation:

(1) $$\zeta =\zeta_{0}\left(\frac{\zeta_{max}{C_{BRI}}^{n}}{K^{n}+{C_{BRI}}^{n}}\right)$$

where *ζ*_0_ is the potential at zero BRI concentration, *ζ_max_* is the asymptote of the curve, *K* is the dissociation constant of BRI for the membrane, and *n* is the Hill coefficient. All experiments were performed in triplicate.

### 2.7. BRI-Induced Permeabilization of Lipid Vesicles

LUVs encapsulating carboxyfluorescein (CF) were prepared from a lipid dispersion formed in a buffer with CF 50 mM (10 mM HEPES, pH 8) [23]. At this concentration, CF is self-quenched. Non-entrapped CF was removed by Size-Exclusion Chromatography (Sephadex G-25, Cytiva, Uppsala, Sweden), and the vesicles were eluted with an elution buffer: HEPES 10 mM + NaCl 186.12 mM to maintain isosmotic conditions with the concentrated CF solution. LUVs (100 μM lipids) were incubated with BRI (0 to 100 μM concentration), and CF fluorescence intensity (ex. 490 nm, em. 513 nm) was assessed after 30 min of incubation using a multiplate reading accessory, FL 6500 Fluorescence Spectrophotometer (PerkinElmer Inc., Madrid, Spain). The released dye percentage was calculated as:

(2) $$CF\%=100\left[\left(I-I_{0}\right)/\left(I_{\infty }-I_{0}\right)\right]$$

where I_0_ is the fluorescence of CF-loaded liposomes, I∞ is the fluorescence after maximal release with Triton X-100 (10 mM). All experiments were performed in duplicate.

### 2.8. Adsorption to the Bare Surface and Insertion into the Lipid Monolayer of BRI

The adsorption of BRI at the air-water interface and its incorporation into preformed lipid monolayers were evaluated using constant-area experiments with a KSV-NIMA minitrough system (Biolin Scientific AB, Västra Frölunda, Sweden). A custom-built trough with a surface area of 3 cm^2^ and a subphase volume of 2 mL was used, as previously described [22]. Adsorption experiments involved injecting increasing volumes of BRI diluted in water into a phosphate-buffered saline (PBS) pH 7.5 or 5 mM HEPES buffer (pH 6.5), as indicated, under continuous stirring. Surface tension (γ) changes were measured using a platinum plate and the Wilhelmy method, while surface pressure (π) was calculated using the equation

(3) $$\left(\pi =\gamma_{0}-\gamma \right)$$

where γ_0_ represents the surface tension of the bare aqueous subphase/air interface, and γ is the equilibrium surface tension after the amphiphile is added.

For insertion experiments, lipid monolayers were formed at the air/aqueous solution interface by stepwise spreading lipids from a chloroform solution (0.2 mM) until the desired (initial) surface pressure was reached. After solvent evaporation, an aqueous BRI solution was injected into the subphase to achieve a final concentration of 40 µM, and the surface pressure was recorded over time until a stable surface pressure was reached (~15 min). All experiments were conducted at 24 ± 1 °C and performed in triplicate.

### 2.9. Langmuir Film Isotherms and Microscopy Visualization

Langmuir lipid monolayers were prepared by spreading appropriate aliquots of chloroform-based lipid solutions onto the aqueous subphase of a Teflon™ trough mounted on a KSV NIMA minitrough system (Biolin Scientific AB, Västra Frölunda, Sweden). After solvent evaporation and film equilibration at a surface pressure π ≤ 0.5 mN/m for approximately 5 min, the monolayers were compressed by symmetrically reducing the area between two Delrin™ barriers at a rate of 6 ± 1 Å^2^·molecule^−1^·min^−1^ until collapse was reached. Barrier movement was electronically controlled and recorded, and surface pressure was monitored as previously described. In parallel, monolayer morphology was visualized using Brewster angle microscopy (BAM). The Langmuir trough was mounted on the stage of a Nanofilm EP3 Imaging Ellipsometer (Accurion, Göttingen, Germany). Minimum reflectance was achieved using a polarized laser (λ = 532 nm) incident on the bare aqueous surface at the experimentally determined Brewster angle (~53.1°). Following monolayer formation and throughout compression, reflected light was collected through a 20× objective and an analyzer-polarized lens and detected with a CCD camera. The gray level of each pixel in the BAM images is related to the monolayer’s film thickness and refractive index [24]. All experiments were performed in triplicate.

### 2.10. Electrochemical Modulation of Black Lipid Membranes by BRI

Black lipid membranes (BLMs) were formed in a polysulfone cuvette containing a 150 µm aperture mounted in a black Delrin chamber (models CF13A-150 and BCH-M13, Warner Instruments Inc., Hamden, CT, USA). Electrical recordings were carried out using a potentiostat/galvanostat (Autolab PGSTAT30) equipped with impedance (FRA) and low-current (ECD) modules. Current and potential data were acquired with NOVA 2.1.2 software (Metrohm Autolab B.V., Utrecht, The Netherlands). The electrochemical cell was configured with a Pt wire counter electrode and an Ag^+^/AgCl/Cl^−^ reference electrode immersed in the cis compartment, and a Pt wire working electrode placed in the trans compartment, as previously described [25].

Aliquots of lipid mixtures (DOPC, M1, or M2) prepared in chloroformic solution were dried under a nitrogen stream and further subjected to vacuum for 1 h to ensure complete solvent removal. The dried lipids were then resuspended in *n*-decane to a final concentration of 20 mg/mL. BLM formation was initiated by pre-coating the aperture from the cis side with 2 µL of the lipid/*n*-decane solution using a micropipette. After solvent evaporation under a N_2_ stream, both compartments were carefully filled with 1 mL of 5 mM HEPES buffer, pH 6.5, which had been previously filtered through a 0.2 µm nylon filter. The aperture was subsequently repainted from the cis side with the lipid solution until bilayer formation, following the protocol described by Corvalán et al. [26].

Bilayer formation was assessed by cyclic voltammetry (potential range ± 50 mV with respect to the open-circuit potential, OCP; scan rate 1 mV s^−1^) and by electrochemical impedance spectroscopy performed 30 min after painting (frequency range 0.01–100 Hz, at 0 V vs. OCP). The presence of a BLM was confirmed by a marked decrease in current (from ~10^−8^ A in the absence of a bilayer to ~10^−11^ A in its presence), an increase in resistance from the MΩ range to the GΩ range, and capacitance values between 0.2 and 0.9 µF cm^−2^, depending on the lipid composition [27]. Resistance and capacitance values were obtained by fitting the experimental data to equivalent electrical circuit models for planar bilayers using ZView software (https://www.zview.com/) (Southern Pines, NC, USA) [25,28].

Once bilayer stability was confirmed, chronoamperometry measurements were performed. The bilayer was held at 0 V versus OCP for 300 s, after which step potentials of +25, +50, +75, −25, −50, and −75 mV (vs. OCP) were applied sequentially for 600 s each. Current–potential (i–E) curves were constructed by calculating the difference between the current measured at 800 s after potential application and the current recorded at 300 s at OCP (i = i_800_s − i_300_s) as a function of the applied potential (see Figure S1 in Supplementary Materials).

Following the characterization of planar bilayers composed of DOPC, M1, or M2, 10 µL of an aqueous BRI solution (40 µM) was added to the cis compartment. After a 60 min stabilization period, impedance spectroscopy and chronoamperometry measurements were performed using the same procedures described above. For each membrane composition, three independent experiments were conducted, and the results are reported as mean ± standard deviation. Statistical analysis was performed using a one-way ANOVA test. The chamber and cuvette were cleaned according to the protocol recommended by Warner Instruments, involving multiple washes with 50 mM sodium phosphate tribasic solution, 0.1% (*w*/*v*) HCl, and Milli-Q water.

## 3. Results

### 3.1. BRI Structure and Electrostatic Properties

Brilacidin (BRI) is a small polymer featuring an arylamide backbone functionalized with both cationic and hydrophobic substituents. To date, its detailed structural and electrostatic properties have not been comprehensively characterized. Nonetheless, the mechanism of action of BRI and its interaction with natural anionic membranes are expected to be strongly governed by its amphiphilic character and electrostatic behavior in aqueous environments. These features may give rise to a well-defined molecular dipole moment, which is likely to control its interaction with the electrostatic field and the electrical double-layer potential at anionic membrane surfaces. To investigate these aspects, Density Functional Theory (DFT) calculations were performed to examine the molecular geometry and the distribution of ionic species as a function of pH, enabling the identification of the most stable conformations and their corresponding protonation states.

DFT is a widely employed quantum-mechanical modeling framework in physics and chemistry for determining the electronic structure of atoms and molecules. DFT commonly relies on a continuum solvent description in which the explicit solvent structure is neglected. A key advantage of this approach is that it enables a quantitative treatment of the solute’s electronic distribution, thereby allowing the assessment of polarization effects at relatively low computational cost [29].

Our results show that BRI is a flexible, cationic amphipathic molecule with an exceptionally large molecular dipole moment (μ = 58.96 D) at pH 6.5, indicating a pronounced charge separation along its molecular backbone (Figure 1a,b). This strong dipole originates from the coexistence of multiple protonated amine groups with hydrophobic aromatic and fluorinated moieties. For comparison, amphipathic α-helical antimicrobial peptides typically contribute ~3.5 D per peptide unit; thus, BRI displays a dipole moment comparable to that of an α-helical peptide of approximately 17 residues. Such electrostatic features are known to modulate peptide interactions with lipid interfaces, as well as their orientation and mechanisms of action [30]. At pH 6.5, the +4 charged species predominate, whereas increasing the pH leads to the population of lower-charge states, with a substantial fraction of the +3 species coexisting with the +4 form at pH 7.5 (Figure 1c).

To the best of our knowledge, this study provides the first DFT-based description of the three-dimensional structure, charge distribution, and dipole moment of BRI under defined protonation conditions, thereby offering a physicochemically grounded framework for interpreting its membrane-related behavior. Notably, DFT–Polarizable Continuum Model (PCM) methodologies have previously been successfully applied to arylamide building blocks used as antimicrobial foldamer monomers, including solvent-screened conformational analyses and the prediction of NMR parameters [31].

BRI displays weak intrinsic fluorescence in aqueous solution (Figure 1d), which is primarily attributed to its conjugated aromatic framework, consisting of substituted phenyl rings and a pyrimidine core that together function as a delocalized chromophore. The CF_3_ group is expected to modulate the electronic energy levels of this system, but does not act as an independent chromophore. Aryl–pyrimidine chromophores with comparable conjugation have been shown to exhibit strong π–π* absorption bands in the 280–340 nm range [32].

Figure 1d shows that BRI exhibits nearly negligible intrinsic fluorescence in pure water, most likely due to efficient quenching by its multiple ionized groups in the aqueous environment. In contrast, a marked increase in fluorescence intensity is observed in apolar media, as illustrated by the spectra obtained in hexadecane. In protic organic solvents such as methanol and isopropanol, the chromophore exhibits a blue shift of the emission maximum relative to hexadecane (Figure 1d). Notably, despite the very similar dielectric constants and dipole moments of methanol and ethanol (Table S1, Supplementary Materials), ethanol does not induce this blue shift, instead displaying a dominant emission peak aligned with that observed in hexadecane. This behavior may be attributed to excimer formation involving the aromatic rings of the fluorophore moiety, which stabilizes the excited state and results in a broadened, red-shifted emission band compared with that of the isolated fluorophore [33]. These solvent-dependent changes in emission wavelength and intensity underscore the sensitivity of the phenyl–pyrimidine moiety to local polarity and microenvironmental effects.

### 3.2. Relevance of Ergosterol in BRI Activity

**Figure 2:**
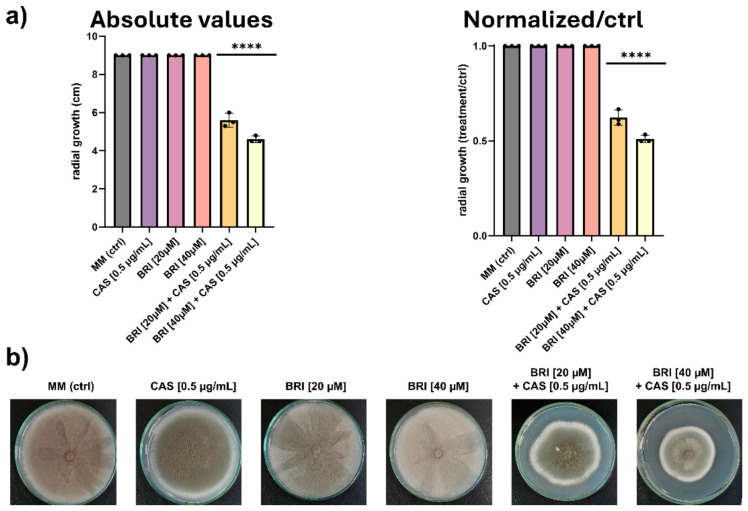
Synergistic inhibitory effect of BRI and CAS on *A. fumigatus*. (**a**) Absolute and normalized growth of *A. fumigatus* in minimal medium (MM, grey bars) in the presence of CAS (violet barrs), BRI (20 μM, lilac bars and 40 μM, pink bars), or the combination of both drugs (CAS 0.5 μg/mL + 20 μM, yellow bars or 40 μM, white bars). (**b**) Representative images of *A. fumigatus* cultures under the corresponding conditions. **** Statistical analysis was performed using a one-way ANOVA with Dunnett’s multiple comparisons test.

BRI acts synergistically with caspofungin (CAS) against both CAS-sensitive and CAS-resistant isolates of *Aspergillus fumigatus*, *Candida albicans*, *C. auris*, and the intrinsically CAS-resistant *Cryptococcus neoformans*, and it also potentiates the activity of azoles against *A. fumigatus* and several Mucorales species [10]. CAS is an echinocandin antifungal drug used to treat severe fungal infections caused by *Candida* and *Aspergillus*. Figure 2 illustrates the synergistic inhibitory effect of BRI and CAS on the growth of *A. fumigatus*.

In *A. fumigatus* and several Mucorales, BRI also enhances the fungicidal activity of voriconazole (VOR) and posaconazole, exhibiting synergistic activity with azoles in vitro [10]. Against *A. fumigatus*, CAS is a fungistatic drug inhibiting β-1,3-glucan synthase non-competitively at the plasma membrane–cell wall interface, while azoles are fungicidal drugs disrupting ergosterol (ERG) biosynthesis and membrane organization. BRI can potentiate CAS against *A. fumigatus*, converting CAS into a fungicidal drug (Figure 2) [10]. Notably, voriconazole (VOR)-resistant *A. fumigatus* isolates that have mutations in *erg11A* (either in the promoter and/or coding regions) have also shown synergy with BRI+VOR [6].

Together, CAS/azole synergism supports a membrane-centric mechanism for BRI, in which sterol content and organization modulate both its intrinsic activity and its capacity to potentiate partner drugs. To further investigate BRI–membrane interactions, we designed a lipid mixture that mimics the plasma membrane of filamentous fungi [21]. This model membrane contains 8.3 mol% phosphatidic acid (PA) within a phospholipid matrix, representing the anionic lipid fraction characteristic of fungal plasma membranes. In addition, PA has been identified as an important regulator of defensin antifungal activity [7,34].

**Figure 3:**
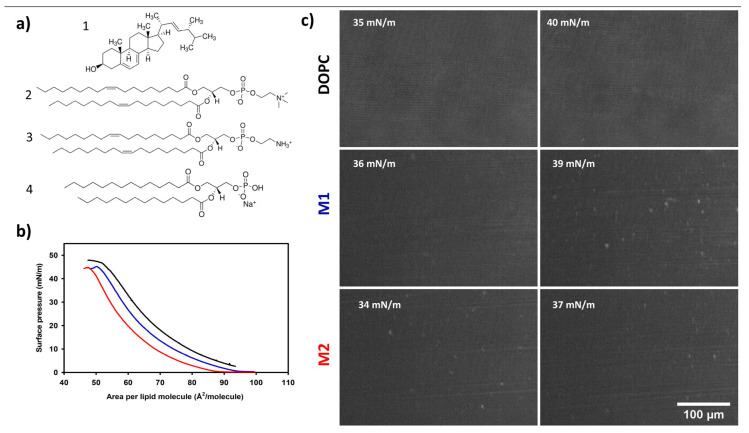
Fungi-inspired lipid model membranes. (**a**) Chemical structures of major fungal membrane lipids: (1) ergosterol (ERG), (2) 1,2-di-(9Z-octadecenoyl)-*sn*-glycero-3-phosphocholine (DOPC), (3) 1,2-di-(9Z-octadecenoyl)-*sn*-glycero-3-phosphoethanolamine (DOPE), and (4) 1,2-ditetradecanoyl-*sn*-glycero-3-phosphate (DMPA; anionic). (**b**) Compression isotherms of DOPC (black) and lipid membranes M1 [DOPC/DOPE/DMPA/ERG (46.3:37:8.3:8.3)] (blue) and M2 [DOPC/DOPE/DMPA/ERG (14.7:33.4:8.3:16.7)] (red), which differ mainly in ERG content. Curves correspond to representative experiments. (**c**) Brewster angle microscopy images of representative monolayers at surface pressures between 30 and 35 mN/m. All experiments were performed in triplicate.

To assess the influence of sterol content on BRI–membrane interactions, ERG was incorporated into the model membranes at 8.3 mol% (M1) and 16.7 mol% (M2) (see Section 2.4 and Figure 3a).

The selected lipid mixtures were characterized using Langmuir monolayer compression isotherms. For unsaturated phospholipid monolayers such as DOPC, compression leads to only a modest increase in surface pressure (π), indicative of a liquid-expanded phase (Figure 3b) [24]. The mixed monolayer M2, containing 16.7 mol% ERG, exhibits similar behavior but at smaller molecular areas, in agreement with previous reports [21] and reflecting the smaller cross-sectional area of sterols compared to phospholipids. As expected, the M1 monolayer, with 8.3 mol% ERG, displays intermediate molecular areas between those of pure DOPC and M2.

Brewster angle microscopy images show that DOPC monolayers are homogeneous, consistent with a purely liquid-expanded phase (Figure 3c). In contrast, M1 and M2 monolayers display less homogeneous surfaces, with small, brighter lipid domains appearing at surface pressures of 35–40 mN/m, a range relevant for the insertion of membrane-active compounds into lipid bilayers. This behavior reflects a more complex membrane organization [24], arising from the increased compositional complexity of the mixed lipid systems.

### 3.3. BRI Surface Activity and Interaction with Model Lipid Membranes

Several studies describe BRI as a defensin-mimetic compound with an amphiphilic architecture [9]. This amphipathic character is thought to originate from the arylamide foldamer scaffold, which segregates cationic and hydrophobic groups onto distinct molecular faces. The guanidinium-like cationic groups are expected to orient preferentially toward the aqueous phase. In contrast, the aromatic phenyl rings and the trifluoromethyl substituent create a nonpolar surface capable of inserting into lipid bilayers. In this configuration, the dipole potential of BRI (Figure 1b and Video S1 in the Supplementary Materials File) would oppose the dipole field generated by the ordered lipids in the proximal membrane hemilayer. This conformational design, reminiscent of host defensin peptides, is therefore likely to promote efficient membrane association [1].

**Figure 4:**
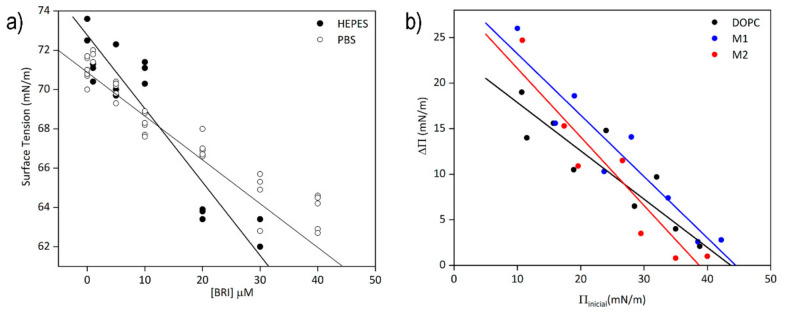
Surface activity and monolayer insertion of BRI. (**a**) Surface tension measurements of BRI at the buffer/air interface. The subphase consisted of PBS at pH 7.5 (open symbols) or 5 mM HEPES buffer at pH 6.5 (closed symbols), at 22 ± 2 °C. (**b**) Insertion of BRI into monolayers composed of DOPC (black), M1 (blue), or M2 (red). The lines represent linear fit of the data. BRI concentration: 40 μM. Cutoff pressures were: DOPC: 44 ± 3; M1: 44 ± 3; M2: 39 ± 2. Error values were estimated from the 95% confidence intervals of the linear regression analysis extrapolated to the abscissa origin.

Amphipathic (or amphiphilic) molecules can partition into anisotropic environments, such as membrane interfaces or the air–water interface, due to their dual hydrophilic–hydrophobic character. Consequently, these molecules behave as surfactants and display surface activity [35]. Adsorption experiments with BRI (Figure 4a) show that adding BRI to the PBS solution leads to a modest reduction in surface tension, with saturation at concentrations near 40 µM. PBS is commonly employed as an experimental medium because its pH (7.2–7.4) and ionic strength are close to physiological conditions.

However, we observed that BRI solubility is strongly dependent on ionic strength, with the compound precipitating as a white pellet in 1 M NaCl. This observation prompted us to examine whether the relatively weak surface activity of BRI in PBS reflects its limited solubility under these conditions. Figure 4a shows that BRI displays only a slight increase in surface activity in a low-ionic strength buffer at pH 6.5, which approximates the acidic environment of alveolar fluid during infection [36]. Under these conditions, BRI reaches saturation of surface activity at 30 µM but, similar to PBS, it does not reduce the surface tension below 62 mN/m at concentrations up to 100 µM. These findings indicate that BRI behaves as a weak surfactant in aqueous media. For comparison, Defensin A and the plant-derived defensin fragment SmAPα1–21 reduce surface tension to approximately 62–55 mN/m [35,37], the bactericidal Polybia-MP1 peptide reaches ~44 mN/m [38], and the antibiotic peptides Maculatin and Citropin, derived from Australian tree frogs, lower surface tension to 49 and 46 mN/m, respectively [39].

BRI interactions with lipid monolayers were investigated using membrane insertion experiments. These were carried out by injecting 40 µM BRI into the aqueous subphase (5 mM HEPES, pH 6.5) beneath a preformed lipid monolayer, prepared by spreading a lipid solution at the air–water interface. Surface pressure is defined as the difference between the surface tension of the bare air–water interface and that of the interface covered by the adsorbed film (Equation (3)) [35]. Upon BRI addition, the surface pressure increased for both anionic and zwitterionic monolayers (Figure 4b). These findings indicate that BRI interacts more favourably with lipid monolayers than would be predicted from its low intrinsic surface activity. Thus, an anisotropic interface such as the bare air–water interface does not provide a sufficiently favourable environment for BRI adsorption. In contrast, the presence of a lipid membrane, which possesses an intrinsic dipole moment, promotes a stronger incorporation of BRI molecules, reflecting an important electrostatic component.

At initial surface pressures below 30 mN/m, the anionic monolayers M1 and M2 exhibited a higher increase in surface pressure than pure DOPC monolayers after BRI insertion, consistent with enhanced recruitment of the cationic BRI by negatively charged membranes. Similar behavior has been reported for the antimicrobial peptides Maculatin and Citropin, which induced increases in surface pressure of approximately 15 mN/m in anionic monolayers but showed minimal insertion into zwitterionic membranes [39]. A comparable sensitivity to surface charge has been observed for Polybia-MP1 [38] and other synthetic antimicrobial peptides, correlating with their antimicrobial and hemolytic activities [40].

Figure 4b also shows that lipid monolayers exclude BRI at surface pressures of 38–45 mN/m, following the order DOPC = M1 > M2. Notably, only the membrane containing 16 mol% sterol displayed a significant limitation to BRI insertion at high surface pressures. This finding suggests that sterol incorporation only modestly modulates membrane susceptibility to BRI insertion. Notably, when phosphate-buffered saline (PBS) was used as the subphase, BRI addition produced only a negligible change in surface pressure, again supporting a strong electrostatic component in BRI insertion into the lipid membranes (Figure 4a).

For AMPs containing at least one tryptophan residue, membrane insertion can be conveniently monitored through shifts in tryptophan fluorescence emission [21]. BRI, however, is an arylamide-based compound that lacks tryptophan but contains several aromatic moieties that are intrinsically fluorescent. As shown in Figure 1d, the fluorescence of BRI is highly sensitive to the polarity of the surrounding medium, reflecting the local environment of its aromatic groups. Environment-sensitive (polarity- or order-sensitive) fluorophores typically display blue shifts and/or changes in emission intensity upon transfer from aqueous environments to the hydrophobic interior of lipid membranes. To assess BRI–membrane interactions, we therefore examined the intrinsic fluorescence of BRI in the presence of an excess of anionic (M1 or M2) or zwitterionic (DOPC) liposomes.

**Figure 5:**
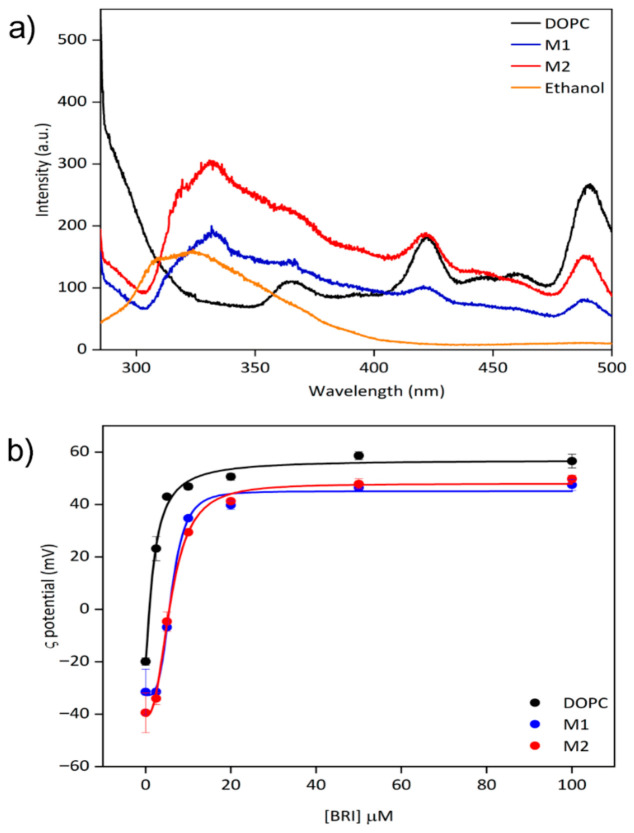
Interaction of BRI with large unilamellar vesicles (LUVs) composed of DOPC or anionic lipid mixtures containing 8.3% (M1) or 16.7 (M2) mol% of ERG. (**a**) Representative intrinsic fluorescence spectra of BRI (40 μM) in the presence of LUVs (excitation wavelength: 266 nm). For comparison, the fluorescence spectrum of BRI in ethanol from Figure 1d is shown in orange. (**b**) ζ potential of liposomes as a function of BRI concentration. Lipid concentration: 200 μM. Curves correspond to DOPC (black), M1 (blue), or M2 (red). The data correspond to the average, and the bars correspond to the SD of triplicate experiments.

Figure 5a shows that, in the presence of DOPC vesicles, BRI exhibits a shoulder in the 280–300 nm region, resembling the emission observed in methanol or isopropanol. This observation is consistent with the monolayer insertion experiments and indicates that BRI interacts with DOPC vesicles by embedding into the membrane interior. In addition, the spectrum shows three further emission bands in the 350–500 nm range, suggesting that BRI populates multiple fluorescent states when bound to liposomes. Thus, membrane binding appears to generate distinct microenvironments, each contributing a separate emission band [41].

Upon measuring BRI fluorescence in the presence of anionic membranes, a new emission band appears at approximately 330 nm, resembling the spectrum observed in ethanol (Figure 5a). This band is significantly more intense for M2 than for M1, suggesting a progressive reorganization of the BRI–membrane configuration with increasing ERG content. The observed red shift may originate from the aggregation of the fluorophore’s aromatic moieties at the anionic membrane surface. At higher surface densities, aromatic fluorophores can form excimers, leading to broadened, red-shifted emission relative to monomer fluorescence [33]. Overall, these findings suggest that BRI interacts differently with zwitterionic and anionic sterol-containing membranes, indicating that membrane composition modulates BRI–membrane interactions, potentially through aggregation-driven processes.

Similar to AMPs, BRI is expected to interact preferentially with anionic cell membranes and their associated ionic double layer, primarily through electrostatic interactions. To further examine the effect of BRI on vesicle surface organization and electrostatics, we monitored the ζ potential, which represents the electric potential at the shear plane of a particle and defines the region that moves under an applied electric field [42].

Liposomes composed of the M1 and M2 mixtures exhibited markedly negative ζ potential values, consistent with their anionic character and with previous reports [21]. Pure DOPC liposomes displayed less negative ζ potentials despite being composed solely of zwitterionic lipids. This behavior arises from preferential ion adsorption at the phosphatidylcholine membrane interface, where strong interactions occur with the zwitterionic choline headgroup [42] (Figure 5b and Table 1).

The addition of micromolar concentrations of BRI resulted in a significant increase in ζ potential for all three liposome compositions, reaching highly positive values at BRI concentrations above 20 µM (Figure 5b). For membrane-active compounds, adsorption onto lipid membranes is often well described by a hyperbolic dependence consistent with a Langmuir-type adsorption model, as observed here for DOPC vesicles. However, this model does not adequately describe BRI adsorption onto M1 or M2 vesicle surfaces. In these cases, the data are better fitted by a sigmoidal function, indicative of cooperative membrane binding (see Equation (1) in Section 2.6 and Table 1).

This cooperative behavior suggests the presence of BRI–BRI interactions at the membrane surface, possibly arising from dimerization or from BRI-induced alterations of the lipid bilayer that generate a higher-affinity membrane state. Similar cooperative membrane adsorption phenomena have been reported for α-synuclein, a protein known to aggregate at membrane surfaces [43]. For all membrane compositions, the BRI concentration required to achieve half of the maximal increase in ζ potential lies at ≤6 µM, indicating a high membrane affinity. Notably, BRI shows an unexpectedly strong affinity for zwitterionic phospholipid vesicles, yielding dissociation constants (K) even lower than those observed for anionic membranes (Table 1). As a reference, BRI exhibits antifungal activity against *C. neoformans* at low concentrations, with a minimal inhibitory concentration (MIC) of 2.5 µM [11], which falls within the range of the dissociation constants measured here. In contrast, BRI displays a much higher MIC (>80 µM) against *A. fumigatus* [10], suggesting a more complex mechanism of interaction with these fungal cells.

### 3.4. BRI Modulates the Electrochemical Behaviour of Model Lipid Membranes

Upon stabilization and insertion into the membrane, AMPs initially cause localized perturbations that can evolve into extensive membrane damage, ultimately leading to bacterial death once a critical surface concentration is reached [44]. Fluorescence-based leakage assays have therefore been widely employed to investigate peptide-induced membrane permeabilization [23,44].

To evaluate BRI’s ability to induce membrane leakage, we performed experiments using self-quenched carboxyfluorescein (CF) encapsulated in lipid vesicles of both anionic and zwitterionic composition. Under all tested conditions, BRI failed to induce consistent vesicle content leakage (Figure S1 in Supplementary Materials), even at high concentrations (up to 100 µM; lipid/BRI ratio of 1). However, at low BRI concentration (6–15 μM), a modest fluorescence leakage (>20%) was detected. This effect may be associated with aggregation of soluble BRI molecules under high ionic strength conditions at elevated concentrations. Dynamic light scattering measurements further confirmed that BRI did not promote vesicle aggregation. These findings are consistent with the absence of detectable membrane insertion of BRI under high ionic-strength conditions.

CF leakage assays require a high intravesicular concentration of the water-soluble fluorophore to achieve self-quenching, which in turn necessitates a high salt concentration in the external medium to preserve isosmotic conditions across the membrane. As a result, although this method is a classical and widely used approach to study membrane permeabilization, it is not suitable for investigating the mechanism of action of BRI, which primarily acts under low ionic strength conditions. In contrast, experiments using *C. neoformans* KN99a cells have shown that BRI increases membrane permeability to propidium iodide (PI) at concentrations of 1.25–25 µM [11]. This observation suggests that BRI may modulate membrane permeability through more subtle mechanisms rather than directly disrupting membranes.

To further explore this possibility, we employed several electrochemical techniques to examine the effect of BRI on ion permeation across free-standing lipid bilayers using the black lipid membrane (BLM) model. BLMs consist of planar lipid bilayers formed across a small aperture separating two aqueous compartments (see Section 2.10). They are typically prepared by “painting” a solution of lipids dissolved in an organic solvent over a hydrophobic aperture, where the lipids self-assemble into a thin bilayer a few nanometers thick. BLMs provide a well-defined and electrically accessible model of biological membranes and are widely used to study ion channels, transport processes, and membrane biophysics under highly controlled conditions [25,26].

Lipid bilayers were evaluated by chronoamperometry and electrochemical impedance spectroscopy (EIS) in both the absence and presence of BRI. EIS has previously been employed to assess the effects of drugs on phospholipid bilayers at physiologically relevant concentrations [45]. This technique is highly sensitive to changes in membrane structure, thickness, and permeability. It has been shown to detect modest yet significant decreases in membrane resistivity consistent with drug adsorption or penetration.

**Figure 6:**
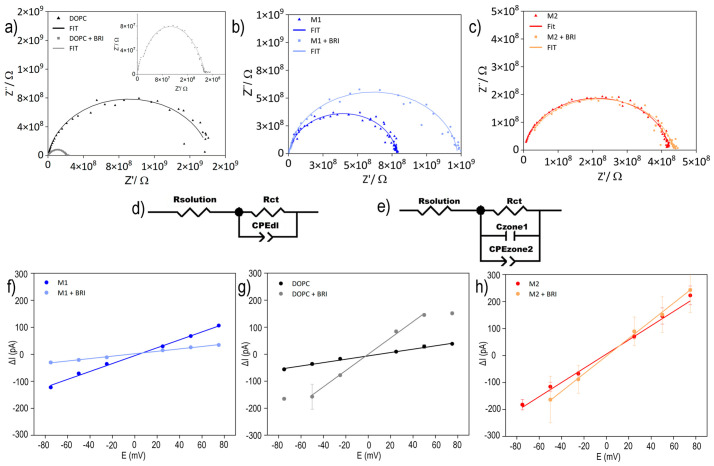
Electrochemical characterization of BRI interaction with black lipid membranes (BLM). (**a**–**c**) Nyquist plot (imaginary vs. real impedance) of BLM in 5mM HEPES, pH 6.5, before (tblack, blue and red symbols) and after (light blue, gray and orange symbols) addition of 0.4 μM BRI for 1 h: (**a**) DOPC (black and grey symbols), (**b**) M1 (blue and light blue symbols) and (**c**) M2 (red and orange symbols). Experimental data were fitted using EIS models (solid lines) corresponding to the Randles equivalent circuit for DOPC, M1, M1 + BRI, M2, and M2 + BRI (**d**) and the pore circuit for DOPC + BRI (**e**). Measurement were performed in the 0,01–100 Hz frequency range with a 5 mV amplitude at open-circuit potential (OCP). (**f**–**h**) Chronoamperometry results showing current change as a function of the applied potential relative to OCP, before (black, blue and red symbols) and after (light blue, gray and orange symbols) addition of 0.4 μM BRI; (**f**) DOPC, (**g**) M1, and (**h**) M2.

EIS perturbs an electrochemical system at equilibrium or steady state by applying a small-amplitude sinusoidal potential (*ac* voltage) over a broad frequency range, while monitoring the resulting sinusoidal current response [46]. Impedance represents the total opposition to alternating current flow in an electrical circuit. For lipid membranes, it is typically modeled as a combination of resistive (R) and capacitive (C) elements. A major advantage of EIS is the ability to fit experimental impedance spectra to equivalent electrical circuits, allowing quantitative extraction of circuit parameters. Impedance data are commonly represented as Nyquist plots, which ideally appear as semicircles when the system is described by a resistor and a capacitor connected in parallel (Figure 6a–e). The charge-transfer resistance (Rct) can be estimated directly from the maximum value of the imaginary component of impedance (*y*-axis).

From impedance results in Figure 6 and Table 2, it can be observed that the capacitance of DOPC bilayers (0.7 µF/cm^2^) is notably higher than that of M1 and M2 bilayers (approximately 0.2 µF/cm^2^). This difference may be attributed to variations in bilayer thickness associated with the presence of sterols. However, although bilayer thickening is a well-established effect in cholesterol-containing membranes, ERG does not appear to significantly increase the thickness of unsaturated phospholipid bilayers [47], exerting only a moderate effect on membranes composed of saturated phospholipids. Therefore, other membrane properties, such as membrane heterogeneity, may cause this difference.

For DOPC BLMs, EIS analysis reveals a resistance of approximately 1.6 × 10^9^ Ω, which decreases to one-eighth of its value (2 × 10^8^ Ω) following the addition of BRI. This change is evident in the Nyquist plot as a reduction in the maximum *y*-axis value (Figure 6a). These results indicate that BRI strongly reduces the planar bilayer’s resistance to ion transport, which may arise from bilayer expansion or the formation of small, conductive defects or pores. A similar effect was observed with the antimicrobial peptide Gramicidin on BLMs, demonstrating its pore-forming capacity [27]. Notably, impedance spectra recorded 1 h after BRI addition cannot be adequately fitted with a simple Randles circuit (Figure 6d). Instead, an additional capacitive element in parallel is required (Figure 6e). This behavior suggests the emergence of two membrane regions (an average membrane and a pore membrane) with distinct conductivities and capacitances (Table 2).

A complementary analysis of DOPC BLMs was performed using chronoamperometry. In this technique, the electrode potential is held constant, while the resulting current is monitored as a function of time (Figure S2), allowing quantification of the permeation of electroactive species under steady-state conditions. Molecules that interact with or permeate the lipid bilayer can alter membrane conductivity and/or structural integrity. The i–E curves for DOPC bilayers (Figure 6f) show a significant increase in conductance (slope) in the presence of BRI compared to DOPC alone, in agreement with the decrease in membrane resistance observed by EIS (Figure 6a and Table 2).

In contrast, when M1 forms the planar bilayer, the presence of BRI leads to the opposite behavior. A more than threefold increase in resistance (corresponding to a decrease in conductivity) is observed in the impedance spectra, from 6 × 10^8^ to 2 × 10^9^ Ω (Figure 6b). This effect is also reflected in a significant reduction of the conductance, as indicated by the lower slope of the i–E curves (Figure 6g). This behavior is consistent with a different type of interaction between BRI and M1 than that previously observed for DOPC membranes, which may involve bilayer compaction and consequently increases resistance to ion transport. Notably, these changes occur without detectable modifications in bilayer capacitance.

For BLMs formed from M2, the addition of BRI does not affect the conductance, as evidenced by statistically equivalent i–E slopes, nor does it alter the resistance measured by impedance spectroscopy (Figure 6c,h and Table 2). These results suggest that the interaction between BRI and M2 does not induce membrane remodeling or structural changes that could affect the bilayer’s electrochemical properties.

It is worth noting that BLMs formed from mixed M1 and M2 lipid compositions exhibit a pronounced reduction in charge-transfer resistance compared to pure DOPC membranes (Table 2). This behavior likely reflects the more heterogeneous and structurally complex nature of mixed membranes, which are intrinsically less resistant than homogeneous, defect-free DOPC bilayers. This is in accordance with the membrane in-plane structure observed in Brewster angle microscopy images of the corresponding monolayers (Figure 3c). In the case of M1, the addition of BRI restores the membrane resistance to values comparable to those of homogeneous DOPC bilayers. This effect is not observed for M2, likely due to reduced structural flexibility and limited reorganization capacity in membranes with high ERG content.

## 4. Discussion

BRI is a synthetic polymer designed to mimic the electrostatic and amphiphilic features of defensin-type antimicrobial peptides. Despite this conceptual framework, no previous studies have reported the minimized structure, electrostatic properties, or surface activity of BRI or related arylamide-based polymers to support this analogy. Our computational analyses show that BRI is a flexible polymer capable of adopting conformations that segregate a highly charged, hydrophilic cationic surface from a hydrophobic one, thereby generating a strong molecular dipole moment. This pronounced amphiphilic dipole is likely to underlie BRI’s strong interactions with both zwitterionic and anionic lipid membranes. In addition, we report the ionization states of BRI over a broad pH range, highlighting the predominance of the +4 charged species at pH 7 and below.

BRI exhibits a strong sensitivity to ionic strength. Its solubility is minimal in 1 M NaCl, and it displays poor surface activity and negligible membrane affinity in PBS, a medium that mimics the pH and osmolality of animal tissues. Reducing the salt concentration markedly enhances BRI’s membrane interaction, consistent with an electrostatically driven mechanism. A similar decrease in the minimum inhibitory concentration of antimicrobial peptides with decreasing ionic strength has been previously reported [48]. How this dependence on ionic strength influences BRI activity across different infection environments remains to be investigated.

BRI is a potent antifungal agent that disrupts membrane potential and permeability. BRI exhibits synergistic activity with azole antifungals, which target ERG biosynthesis, further supporting a membrane-associated mechanism of action [10,11]. In the present study, we examined BRI interactions with model lipid membranes to gain mechanistic insight into its antifungal activity. Two distinct behaviors were observed depending on membrane composition, namely homogeneous zwitterionic phospholipid membranes and anionic membranes containing ERG.

In DOPC membranes, BRI behaves similarly to AMPs. It exhibits strong adsorption to DOPC monolayers, even at high initial surface pressures, indicating a marked tendency to insert into the lipid film. This insertion is supported by an increase in intrinsic fluorescence intensity, with emission features comparable to those observed in certain protic organic solvents. Consistently, Langmuir-type adsorption of BRI to DOPC vesicles was confirmed, revealing high affinity and the development of a large positive ζ potential. Moreover, the accumulation of BRI within DOPC membranes reduces membrane resistance to ion permeation and increases membrane conductance, consistent with a pore-forming mechanism. Together, these observations highlight the similarity between BRI and the canonical mode of action of AMPs, where pore formation and enhanced membrane permeabilization are hallmark features [1,44].

A markedly different behavior was observed when BRI interacts with anionic membranes containing ERG, which more closely mimic fungal cell membranes [21,49]. At low initial surface pressures, BRI insertion into anionic monolayers produced a greater increase in surface pressure than in zwitterionic DOPC membranes, suggesting deeper or more extensive insertion, as expected from electrostatic considerations. However, at high surface pressures—within the range relevant for bilayer membranes (35–40 mN/m)—the presence of 16 mol% ERG appeared to hinder BRI insertion. This effect was dependent on ERG content and likely reflects the increased membrane compactness [50] and resistance to incorporation of membrane-active compounds conferred by ERG, as commonly observed for other sterols [24,51].

Additional insight into BRI interactions with fungi-mimicking membranes was obtained from intrinsic fluorescence measurements. Our studies revealed a broad emission band centered at 320–340 nm, red-shifted relative to that observed in zwitterionic DOPC vesicles. This emission resembles that observed in apolar solvents and suggests the formation of excimers involving the fluorophore groups. The intensity of this band was also dependent on ERG content, implying a distinct BRI conformation in these membranes, likely associated with dimer formation. Supporting this interpretation, ζ-potential versus BRI concentration curves showed a clear sigmoidal profile for ERG-containing anionic membranes, consistent with in-plane dimerization or other forms of BRI aggregation at the membrane interface.

This scenario is further supported by electrochemical data from BRI interactions with BLMs. In the anionic BLM containing 8.3 mol% ERG, the membrane resistance was already lower compared to DOPC membranes. Addition of BRI increased resistance, reducing ion permeation—an effect opposite to what is seen in DOPC systems. Notably, the BRI effect disappeared when ERG content was increased to 16.7 mol%, similar to native fungal membranes. These results strongly suggest that BRI acts through a different mechanism than the pore-forming behavior seen in zwitterionic phospholipid membranes and typical AMPs. Importantly, BRI’s sensitivity to membrane ERG content provides a mechanistic link to the reported synergy between BRI and azoles [10], a drug class that disrupts ERG biosynthesis and ultimately lowers ERG levels in fungal membranes.

ERG, similar to cholesterol, promotes the formation of lipid domains in phospholipid membranes depending on concentration [50]. We confirmed this in our lipid systems using Brewster angle microscopy to image monolayers. Incorporating membrane-active compounds into heterogeneous membranes can shift phase balances, especially when they have preferential interactions with one phase [52]. Such shifts toward the expanded or disordered phase have been repeatedly observed for amphiphiles capable of penetrating lipid bilayers [22,24,52].

In our systems, homogeneous zwitterionic BLMs exhibited a high resistance to ion permeation. By contrast, mixed BLMs containing domains showed a markedly lower resistance, indicating higher conductance. This difference may arise from enhanced ion permeation near lipid domains, where structural defects are more likely to form. In membranes with lower ERG content, BRI restored the high resistance characteristic of homogeneous membranes, possibly by shifting phase equilibria toward a continuous liquid-disordered phase and thereby promoting membrane uniformity. This effect was not observed at higher ERG levels, where the membrane likely remains outside the homogeneous region of the phase diagram even after interaction with BRI.

Our results highlight distinct aspects of drug–membrane interactions, showing that subtle changes in membrane properties—such as lateral membrane organization (domain formation/disruption), membrane thickness, and ion permeability—can be differentiated from more extensive effects like pore formation or membrane disruption. These subtle membrane alterations may also affect other membrane-associated processes, including BRI aggregation/dimerization, which appears to depend on membrane heterogeneity. Additional membrane-associated events may likewise be modulated, potentially compromising cell viability.

## 5. Conclusions

Overall, these results suggest that BRI’s action on complex lipid membranes involves a different mechanism than in simple, homogeneous membranes. This mechanism seems to include BRI–BRI interactions at the membrane surface, potentially through dimerization or aggregation near lipid domains, as reported for other membrane-active compounds [53]. These interactions may influence phase coexistence and lipid domain organization in an ERG-dependent way. Therefore, a mechanism involving ERG-rich membrane domains—closely related to the lipid raft concept—emerges as a plausible mode of action. Taken together, our findings not only link ERG content to BRI activity in a manner consistent with its synergy with azoles but also suggest a broader connection with lipid raft organization in fungal cell membranes. This opens new possibilities for understanding the role of sterol and sphingolipid metabolism in BRI’s mechanism of action, as previously reported [10,11]. Despite providing important mechanistic insights into the behavior of BRI, the present study has several limitations associated with the use of simplified model membrane systems. Although these models are valuable for dissecting specific lipid-dependent interactions, they cannot fully reproduce the compositional complexity and dynamic heterogeneity of biological membranes. Future studies combining microbiological and advanced biophysical approaches will complement the present findings and provide deeper insight into the roles of membrane heterogeneity, lipid raft organization, and sterol-dependent domain modulation in the mechanism of action of arylamide-based foldamers such as BRI. Considering the limited number of antifungal drugs currently available, elucidating the mechanism of action of BRI broadens the prospects for the development of foldamer-based antifungal agents.

## Figures and Tables

**Table 1:** Analysis of liposome surface electrostatics in the absence or presence of BRI.

Lipid Composition	ζ_0_ (mV) BRI (−)	ζ_max_ (mV) BRI (+) *	K (μM)	n (Hill Coefficient)
DOPC	−20 ± 3	57 ± 4	2.0 ± 0.3	1.3 ± 0.4
M1	−33 ± 3	45 ± 3	6.1 ± 0.3	3.7 ± 0.6
M2	−41 ± 2	48 ± 3	5.9 ± 0.3	2.5 ± 0.3

* ζ potential of LUVs was obtained in the absence (ζ_0_) or presence of BRI. Parameters were obtained by adjustment of the data in Figure 5b with a hyperbolic sigmoidal function (Equation (1)). ζ_max_ BRI (+) corresponds to the asymptote value of the fit of triplicate experiments. Lipid concentration: 200 μM.

**Table 2:** Electrochemical parameters of BLMs of different compositions in the absence or presence of 40 μM BRI.

Membrane Composition	Amperometry *	Impedance Parameters **	
	Conductance (Slope) (pA/mV or nS)	C (μF·cm^2^)	Rct (Ω)
DOPC	0.63 ± 0.03	0.69 ± 0.02 Czone 1	(1.6 ± 0.5) × 10^9^
DOPC + BRI	3.0 ± 0.1	1.3 ± 0.7 Czone 1	(2.2 ± 0.3) × 10^8^
		0.17 ± 0.07 Czone 2	
M1	0.89 ± 0.04	0.18 ± 0.04	(5.9 ± 0.8) × 10^8^
M1 + BRI	0.38 ± 0.01	0.17 ± 0.02	(2 ± 1) × 10^9^
M2	2.6 ± 0.1	0.18 ± 0.04	(2.0 ± 0.5) × 10^8^
M2 + BRI	3.2 ± 0.1	0.25 ± 0.06	(2.6 ± 2) × 10^8^

* Conductance obtained from the slope of Figure 6f–h. The r^2^ in the linear range of the I vs. E curve was higher than 0.99 for all bilayers. F-test: DOPC/DOPC_BRI and M1/M1_BRI slopes are statistically different for α = 0.01 (*p*-value < 0.0001), whilst for M2/M2_BRI, there is insufficient statistical evidence to conclude that the slopes are different at α = 0.01 (*p*-value = 0.06). ** Impedance parameters obtained from the fitting result in Figure 6a–c; Rct: transference resistance and C: capacitance value.

## Data Availability

The original contributions presented in this study are included in the article/Supplementary Materials. Further inquiries can be directed to the corresponding author.

## Supplementary Materials

The following supporting information can be downloaded at: https://www.mdpi.com/article/10.3390/antibiotics15060548/s1, Figure S1: Liposome permeabilization assay. (a) Percent of carboxyfluorescein release (CF%; see Equation (2)) from POPC (black) or M2 (red) LUVs, 30min after BRI addition. (b) Raw fluorescence data at 513 nm (ex. 490 nm) of CF loaded liposomes in the absence or presence of BRI at the indicated concentration. 100% release control was assessed by the addition of SDS 17 mM. The final lipid concentration was 100 μM. Error bars represent the SD of two independent experiments; Figure S2: (a) Potential program applied from the OCP for chronoamperometry, (b,c) Current response to the potential stimulus applied in (a) in before and after BRI addition for DOPC BLM. Table S1: Dielectric constants and permanent gas phase dipole moments of organic solvents used in this work.; Video S1: 3D chemical structures of BRI. Geometry optimization and electronic properties (electrostatic charges and molecular dipole moment) were obtained at the DFT level using the B3LYP/6-31+G* method with implicit solvent (PCM, water).
