# Supplementary material for: Brilacidin’s Antifungal Mechanism: Insights from Lipid Membrane Models
Source: Antibiotics (Basel). 2026 May 29;15(6):548. doi: 10.3390/antibiotics15060548 (PMC13295559; doi:10.3390/antibiotics15060548)
Supplement: Supplementary file 1 [file antibiotics-15-00548-s001.zip › antibiotics-4283515-supplementary.pdf]

SUPPLEMENTARY MATERIAL

Brilacidin's Antifungal Mechanism: Insights from Lipid Membrane Models

María Victoria López Nota Francisco, Milagro Mottola, Jessica A. Valdivia Pérez, Julieta Tallone, Thaila Reis, Gustavo H. Goldman, Candelaria I. Cámara, Maria Laura Fanani

Video S1. 3D chemical structures of BRI. Geometry optimization and electronic properties (electrostatic charges and molecular dipole moment) were obtained at the DFT level using the B3LYP/6-31+G* method with implicit solvent (PCM, water).

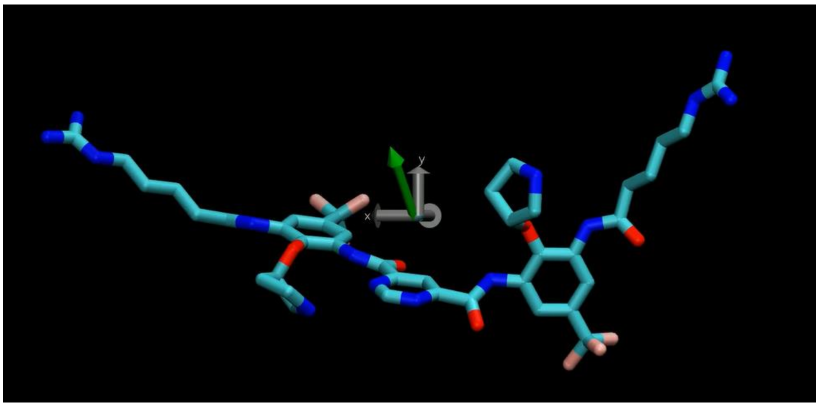

Table S1. Dielectric constants and permanent gas phase dipole moments of organic solvents used in this work.

Solvent	Dielectric constant (≈25 °C)	Dipole moment (D, gas phase)
**Water**	78–80	≈1.85
**Methanol**	32–33	≈1.70
**Ethanol**	24–26	≈1.69
**Isopropanol**	18–20	≈1.66
**Hexadecane**	~2.0–2.1	≈0 D

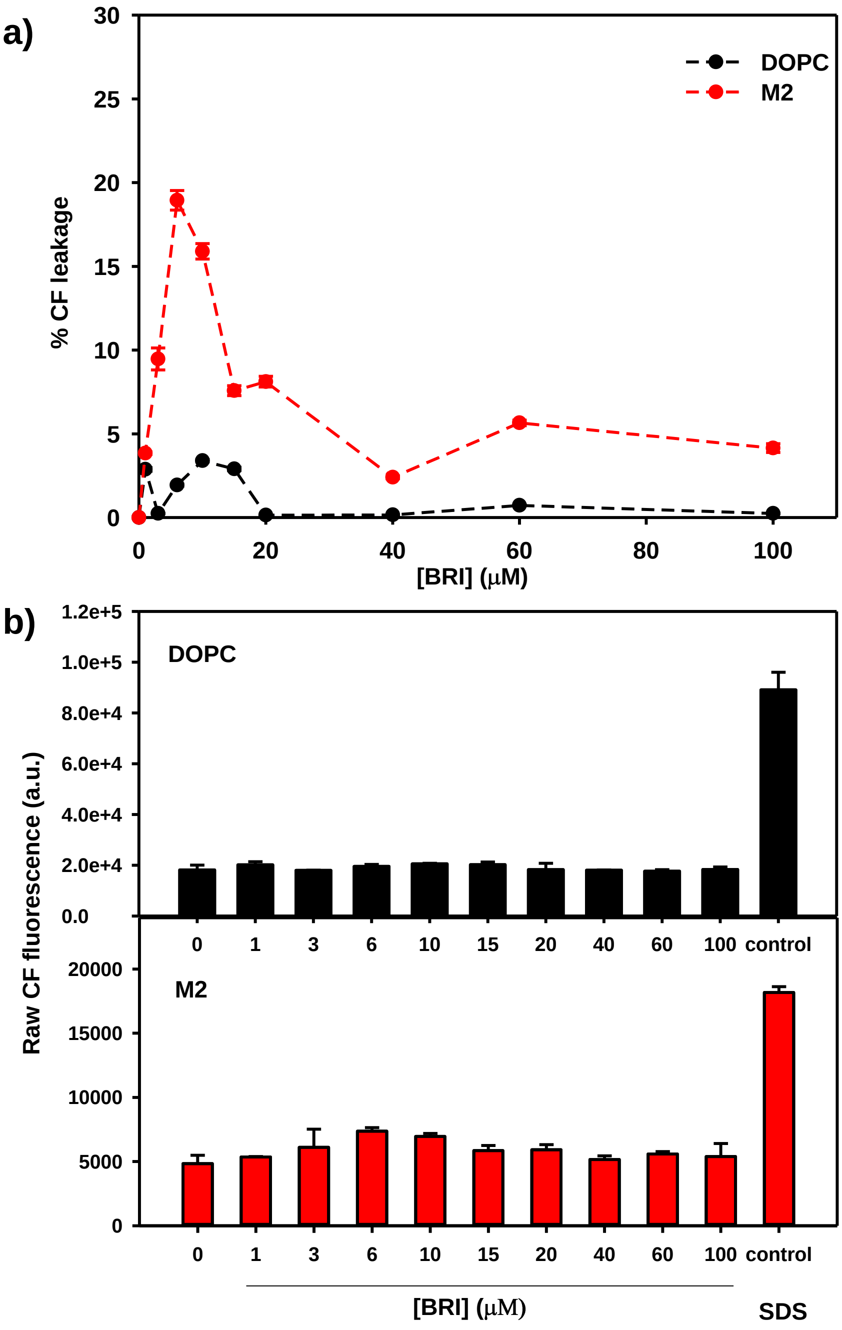

Figure S1: Liposome permeabilization assay. a) Percent of carboxyfluorescein release (CF%; see Eq. 2) from POPC (black) or M2 (red) LUVs, 30min after BRI addition. B) Raw fluorescence data at 513 nm (ex. 490 nm) of CF loaded liposomes in the absence or presence of BRI at the indicated concentration. 100% release control was assessed by the addition of SDS 17 mM. The final lipid concentration was 100 μM. Error bars represent the SD of two independent experiments.

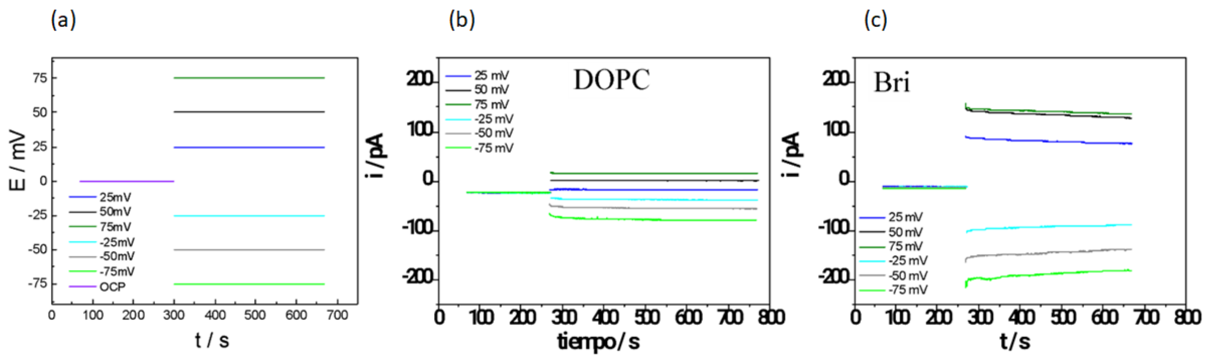

Figure S2: (a) Potential program applied from the OCP for chronoamperometry, (b-c) Current response to the potential stimulus applied in (a) in before and after BRI addition for DOPC BLM.
